# The Turkish Version of the Rhinoplasty Outcomes Evaluation Questionnaire: Validation and Clinical Application

**DOI:** 10.4274/balkanmedj.galenos.2018.2018.1129

**Published:** 2019-02-28

**Authors:** Mustafa Çelik, Ahmet Altıntaş

**Affiliations:** 1 Department of Otorhinolaryngology, Head and Neck Surgery, Kafkas University School of Medicine, Kars, Turkey; 2 Clinic of Otorhinolaryngology, Head and Neck Surgery, Fatih Medical Park Hospital, İstanbul, Turkey

**Keywords:** Quality of life, questionnaire, rhinoplasty, validity and reliability

## Abstract

**Background:**

An assessment of rhinoplasty from the patient’s perspective, in terms of satisfaction and quality of life, is quite important because these are the predominant factors indicating the success of rhinoplasty.

**Aims:**

To translate the Rhinoplasty Outcomes Evaluation into Turkish and then validate the new version for use in Turkish patients.

**Study Design:**

Validation study.

**Methods:**

We enrolled 30 participants who were able to read and write Turkish and underwent primary rhinoplasty. The control group consisted of 58 healthy volunteers with no need for aesthetic or functional nasal surgery. The reliability of the Rhinoplasty Outcomes Evaluation-T was analyzed according to its internal consistency and test-retest reproducibility. Discriminant validity was calculated by comparing the Rhinoplasty Outcomes Evaluation-T scores between the patient and control groups. Responsiveness and sensitivity to changes in rhinoplasty outcomes were analyzed by comparing the patients’ pre- and postoperative Rhinoplasty Outcomes Evaluation-T scores.

**Results:**

The scores for questions 1-6 of the Rhinoplasty Outcomes Evaluation-T, as well as the total scores, were significantly lower in the patient group than in the control group (all p<0.05). In the patient group, the scores for questions 1-6 of the Rhinoplasty Outcomes Evaluation-T, as well as the total scores, were higher postoperatively than preoperatively (all p<0.05). The scores for each Rhinoplasty Outcomes Evaluation-T question, as well as the total scores, did not differ significantly with respect to test-retest reproducibility (all p>0.05). The internal consistency of the Rhinoplasty Outcomes Evaluation-T was high, as evidenced by Cronbach’s α values of 0.887 preoperatively and 0.798 postoperatively.

**Conclusion:**

The Rhinoplasty Outcomes Evaluation-T constitutes a validated instrument with which to measure rhinoplasty outcomes among Turkish patients.

Rhinoplasty (RP) is among the most commonly performed surgeries for both aesthetic and functional purposes (1). Most studies of RP have focused on the surgical technique, level of surgeon experience, development of complications, and revision rate (2,3) However, an assessment of RP from the patient’s perspective, in terms of satisfaction and quality of life, is equally important because these are the predominant factors indicating the success of RP, as well as its ultimate purpose (4,5).

Patient satisfaction with the results of RP is generally lower than with those of other facial aesthetic surgeries (6,7). Among the factors determining patient satisfaction with the RP outcome are the social environment, level of education, life experience, and expectations; the patient’s expectations may not be realistic and may differ markedly from those of the surgeon (8). Furthermore, while for the majority of patients, postoperative function is more important, in other cases function and aesthetics are of equal concern. Understanding the patient’s expectations preoperatively is essential to ensuring satisfactory results. The degree of patient and surgeon satisfaction will show a mismatch if the patient does not recognize the limits of RP in his or her particular case. Consequently, an evaluation of the outcome of surgical success will be difficult.

The information obtained in quality of life questionnaires provides the basis for quantitative assessment of subjective outcomes, including patient satisfaction with the outcome of RP. Although general questionnaires, such as the Short Form-36 health survey questionnaire, are often used to assess the quality of life of RP patients, a disease-specific questionnaire may be more suitable in this context (9).

Therefore, in 2000, Alsarraf (10) created a series of questionnaires with a view to specifically investigating the outcomes of facial aesthetic procedures, including RP, in terms of patient satisfaction. The Rhinoplasty Outcomes Evaluation (ROE) was developed to assess RP outcomes, and comprises six questions (two each for physical, emotional, and social factors) relevant to patient satisfaction. The ROE is widely used and has been translated from the original English into several other languages, including German and Brazilian-Portuguese (2,8). To allow data comparison between new versions of questionnaires and those already available in the literature, careful adaptation of the existing questionnaires is necessary; mere translation is inadequate. Furthermore, newly developed questionnaires must be validated. In this study, given its popularity and utility, we translated the ROE into Turkish and then validated the new version for use in Turkish patients.

## MATERIALS AND METHODS

This study was conducted in accordance with the principles of the Declaration of Helsinki and all applicable regulatory requirements and good clinical practice guidelines. The study protocol was approved by the Ethics Committee of our hospital (Ethics Committee Number: 2018/15). All patients were informed of the study’s purpose and all provided written informed consent. The first step of the present study was the application for authorization to the original researcher.

The ROE-Turkish version (ROE-T) is a forward- and backward-translated version of the ROE (Appendix 1). The ROE was translated and adapted according to the criteria of Guillemin et al. (11) It comprises six questions, each answered on a scale of 0 to 4 where “0” is the most negative and “4” the most positive response. The total score is calculated by adding the scores of the individual questions and therefore ranges from 0 to 24. To facilitate interpretation of the results, the total score can be divided by 24 and multiplied by 100, yielding a percentage value between 0 and 100%, where higher values denote greater levels of patient satisfaction.

We applied the ROE-T in a non-randomized, prospective study performed in the Department of Otolaryngology of our hospital. The sample size was calculated on the basis of a sample size estimation formula for cross-sectional studies (12). Using a 95% level of confidence, powered at 0.8, with a standard size effect size of 0.72, a minimum sample size of 30 for each group was required. We enrolled 30 participants, aged between 18 and 65 years, who were able to read and write Turkish and underwent primary RP. There were 10 males and 20 females, with an average age of 27.40±4.40 years (range: 19-35 years). Patients were excluded if they had congenital facial deformities, were undergoing revision RP, did not speak Turkish, or were unwilling to participate in the study.

The control group consisted of 58 healthy volunteers with no need for aesthetic or functional nasal surgery. They were recruited from among the employees of our hospital, their relatives and students. The 17 males and 41 females comprising the control group had an average age of 28.20±7.80 years (range: 19-47 years).

The ROE questionnaire was completed during three visits. At the first visit (day of enrolment), the questionnaire was completed by the patients and members of the control group. During the second and third visits, the questionnaire was filled out only by the patient group. The second visit took place during the preoperative period, 2 weeks after the first visit, and was designed to assess the reproducibility of the questionnaire scores. The third visit occurred 3 months postoperatively.

### Statistical analysis

Statistical analysis was performed using SPSS software (ver. 22.0; SPSS Inc., Chicago, IL, USA). Descriptive statistics were calculated for all variables, including frequencies and percentages for nominal variables and measures of central tendency (means and medians) and dispersion (standard deviations and ranges) for continuous variables. The Kolmogorov-Smirnov test was used to evaluate the distribution of the data. The significance of each intergroup difference was analyzed using the Student’s t-test, and the significance of any difference in median values was assessed by the Mann-Whitney U test or chi-square test. Quantitative data were analyzed using the Wilcoxon test.

The reliability of the ROE-T was analyzed according to its internal consistency and test-retest reliability. Internal consistency was determined by calculating Cronbach’s α, for which the minimum acceptable score is 0.7 (13). The test-retest reliability, a measure of stability and reproducibility, pertains to the ability of a scale administered on separate occasions to achieve consistent results. Test-retest reliability was calculated by comparing the ROE-T results obtained at the first and second visits (separated by a 2-week interval without treatment) in the patient group.

Discriminant validity was calculated by comparing the ROE-T scores between the patient and control groups using the Mann-Whitney U test and chi-square test. Responsiveness and sensitivity to changes in RP outcomes were analyzed by comparing the patients’ pre- and postoperative ROE-T scores. A p value <0.05 was considered to indicate statistical significance.

## RESULTS

There was no statistically significant difference between the patient and control groups in age or sex (both p˃0.05).

### Discriminant validity of the ROE-T

The scores for questions 1-6 of the ROE-T, as well as the total scores, were significantly lower in the patient group than in the control group (all p<0.05) (Table 1). This result demonstrated the suitability of the ROE-T for identifying patients who are candidates for RP; that is, the ROE-T has acceptable discriminant validity.

### Responsiveness of the ROE-T to changes in RP outcomes

In the patient group, the scores for questions 1-6 of the ROE-T, as well as the total scores, were higher postoperatively than preoperatively (all p<0.05). Thus, the questionnaire was adequately sensitive in terms of detecting changes in patient satisfaction during the postoperative period. The changes in ROE-T scores between the preoperative and postoperative periods are presented in Table 2.

### Reliability of the ROE-T

Test-retest reproducibility was evaluated in the patient group preoperatively to avoid any effect of surgery on the questionnaire scores. Neither the scores for each ROE-T question nor the total scores differed significantly with respect to test-retest reproducibility (all p>0.05; Table 3).

The internal consistency of the ROE-T was high, as evidenced by Cronbach’s α values of 0.887 preoperatively and 0.798 postoperatively.

## DISCUSSION

RP is a common surgical procedure that has a substantial effect on patients’ quality of life, the assessment of which, whether for treatment or research purposes, must be performed accurately. Numerous questionnaires are used to investigate the quality of life of RP patients, including the ROE, which is short and easy to apply and has been applied in several published studies (2,8).

Before a questionnaire can be used in a population other than the one for which it was designed, it must be translated and culturally adapted and its psychometric features evaluated and compared to those of the original version. The ROE has six basic questions, is easy to apply and is appropriate for use by otorhinolaryngologists in the primary care setting. However, until this study, although an unpublished study by Ünlü et al. (14) was previously presented at the Turkish Otorhinolaryngology XXXI National Congress, no version of the ROE was available for Turkish patients. We therefore translated the ROE into Turkish and culturally adapted it to ensure the reliability and validity of ROE data collected on Turkish patients. Alsarraf (10) pointed out the need to preserve the ease of use of the ROE, as well as its easy-to-understand format. Regarding the ROE-T, after translation and cross-cultural adaptation, even patients who had difficulties with reading and comprehension were able to complete the instrument.

The results demonstrate that the ROE-T is a valid and reliable instrument for evaluating outcomes in RP patients. The validity of the ROE-T was shown by its ability to differentiate patients requiring RP from the general population (3.3±2.3 vs 15.8±5.5, p<0.001). These results were consistent with those of previous studies. The good internal consistency of the ROE-T was confirmed by a preoperative Cronbach’s α value of 0.887. The test-retest reproducibility of the ROE-T was also excellent, with the rate being comparable to that previously published in the literature (2,8). Among our patients who underwent RP, the ROE-T scores showed a significant improvement at 3 months after surgery (3.3±2.3 vs 22.2±2.8, p<0.001), providing further evidence of the high responsiveness of the ROE-T to changes in RP outcomes. To the best of our knowledge, the ROE has not been translated into Turkish in any previously published study, nor has it been culturally adapted for use in the Turkish population.

An improvement in the ROE score post- versus pre-RP has been previously reported. Alsarraf et al. (15) reported a mean increase in ROE score of 44.5% among patients who underwent RP, while Izu et al. (2) noted a mean increase of 55.66%, Bulut et al. (8) 21.7%, and Başer et al. (16) 54.26%. In the present study, the average postoperative improvement in the ROE-T score was 75%, from a score of 3.3 (13.75%) preoperatively to 22.2 (92.5%) postoperatively. The greater postoperative improvement in the ROE-T score in our study compared with previous studies may be attributable to the small sample size and the significant improvement in both aesthetic and functional expectations of our patients. The preoperative scores for each question were lower in our study versus previous reports but improved considerably in the postoperative assessment. This result suggests that the surgeon should be aware of both the functional and the aesthetic issues associated with RP.

The follow-up period varies widely among studies in the literature (1,2,5,6,7,8,9). Izu et al. (2) attributed the differences in ROE scores between postoperative day 15 and the third postoperative month to a decrease in postoperative edema at the later time point. The follow-up period is very important in determining the RP outcome, although outcomes may not change over longer follow-up periods. Arima et al. (17) found no difference in quality of life between follow-up periods of 6 months and 10 years. In the present study, we postoperatively evaluated our patients only once, at the third postoperative month, and compared only the pre- and postoperative ROE-T scores. However, our aim was not to evaluate the quality of life after RP but rather to translate the ROE into Turkish and validate the ROE-T in a Turkish population.

The present study had both strengths and limitations. The discriminatory power of the ROE-T was determined statistically based on the difference in scores before versus after RP. However, the minimal clinically important difference should be determined, and the discriminatory power of the ROE tested in clinical practice. By translating the ROE and validating the resultant ROE-T, we have set the stage for further studies on changes in the quality of life of RP patients. However, there were limitations to our study, including the small population size and, potentially, the duration of the follow-up period (where it is unclear whether postoperative ROE-T scores would likely change substantially over longer follow-up periods) (17).

As a conclusion, in this study, the reliability and validity of the ROE-T were demonstrated, and the results were comparable to those of the original ROE. The ROE-T thus constitutes a validated instrument with which to measure RP outcomes among Turkish patients. As the ROE is the best-validated tool for assessing the outcomes of RP, the ROE-T can be applied as part of multi-national investigations.

## Figures and Tables

**Appendix 1:**
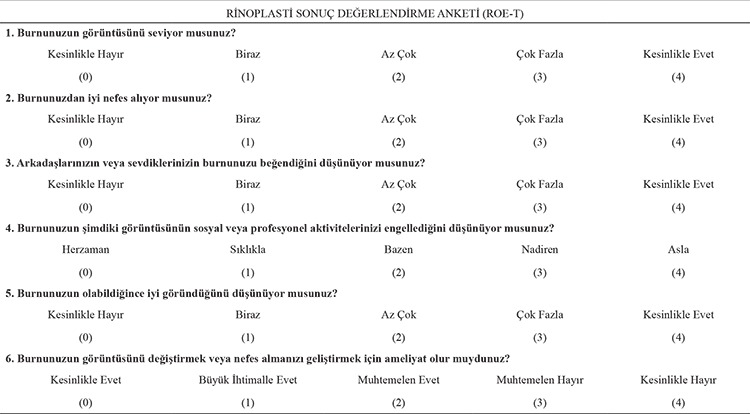
Turkish version of the Rhinoplasty Outcomes Evaluation questionnaire

**Table 1:**
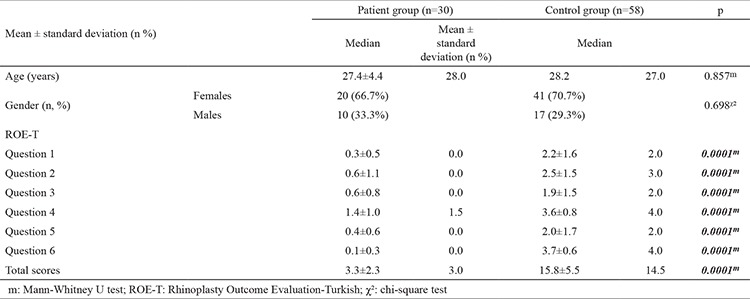
Demographic data and ROE-T results for all tested groups

**Table 2:**
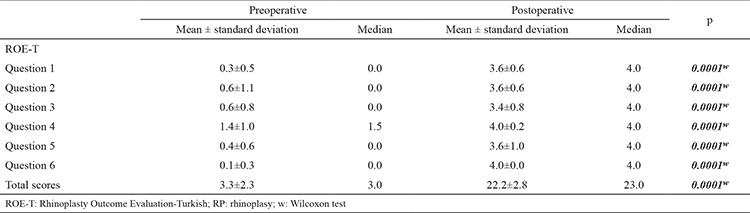
Responsiveness of the ROE-T to changes in RP outcomes

**Table 3:**
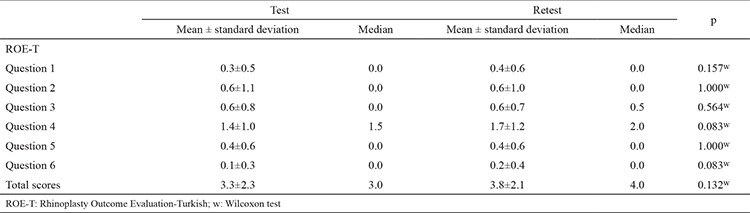
Test-retest reliability of the ROE-T in the patient group
